# Combating Alzheimer’s through sleep and exercise (CASE): A study protocol

**DOI:** 10.1007/s11357-026-02337-1

**Published:** 2026-06-06

**Authors:** Debbie Chung, Brittany A. Larsen, Laurent G. Garchitorena Gomez, Alberto R. Ramos, Neva J. Kirk-Sanchez, Girardin Jean-Louis, Azizi A. Seixas

**Affiliations:** 1 Department of Psychiatry and Behavioral Sciences, University of Miami Miller School of Medicine, 1120 NW 14th St., Miami, FL 33136 USA; 2 Department of Neurology, University of Miami Miller School of Medicine, 1150 NW 14th Street, Miami, FL 33136 USA; 3 Department of Physical Therapy, University of Miami Miller School of Medicine, 5915 Ponce de Leon Blvd., 5th Floor, Coral Gables, FL 33146 USA; 4 Department of Informatics and Health Data Science, University of Miami Miller School of Medicine, 1120 NW 14th St, Miami, FL 33136 USA

**Keywords:** Alzheimer’s disease, Cognition, Exercise, Home-based digital health, Mild cognitive impairment, Sleep

## Abstract

Dementia affects over 55 million people worldwide, with Alzheimer’s disease (AD) comprising most cases. Because curative therapies remain unavailable, there is an urgent need for prevention strategies that target modifiable risk factors. Behavioral interventions—particularly sleep improvement and physical activity—can independently benefit cognition, brain function, and cardiometabolic health, yet their combined (potentially synergistic) effects and scalable delivery models remain underexplored. This exploratory, hypothesis-generating, three-arm randomized controlled trial will evaluate: (1) whether improvements in sleep metrics are associated with improvements in cognitive performance and biomarker panels reflecting brain and cardiometabolic health; (2) the effects of structured, moderate-intensity aerobic and anaerobic exercise on cognitive performance and biomarker panels; (3) the combined effects of sleep and exercise interventions on cognitive performance and biomarker panels, including potential synergy; and (4) the role of light exposure as a modulator of sleep parameters. Over 8 weeks at a single site, 60 community-dwelling adults, aged 55–80 years, with and without mild cognitive impairment, will be randomized to exercise-only, sleep-only, or combined sleep-plus-exercise interventions. Programs will be delivered in-person and/or using Elemind Neuromodulation headbands, Fitbit trackers, and Lys light sensors. Cognitive function will be assessed with the Digital Neuro-Signature Platform, and plasma biomarkers will index cardiometabolic health and AD-related neuropathology. Findings will inform larger, multi-site trials targeting modifiable AD risk factors using scalable, digital, and decentralized intervention platforms. ClinicalTrials.gov NCT04855630; https://clinicaltrials.gov/study/NCT04855630. Registration date 04/19/2021.

**Supplementary Information:**

The online version contains supplementary material available at https://doi.org/10.1007/s11357-026-02337-1.

## Introduction

### Background and rationale

Dementia currently affects over 55 million people globally, with projections rising to 139 million by 2050 [1]. Alzheimer’s disease (AD), the most common form of dementia, composes 60–80% of all dementia cases globally [2]. As no curative treatments exist to date, research has increasingly focused on modifiable lifestyle-based strategies that may delay or prevent the onset of mild cognitive impairment (MCI), a prodromal stage of AD. Two such promising modifiable lifestyle-based strategies include sleep and exercise. High-quality sleep, particularly adequate slow-wave sleep (SWS) and rapid eye movement (REM) sleep, has been associated with improved cognition in older adults, especially those with cognitive deficits [3, 4]. Similarly, exercise has been shown to improve cognitive function and preserve hippocampal volume, a decrease of which serves as a prodromal neurobiomarker of AD risk, although the precise biological mechanistic underpinnings to elucidate this remain under investigation [5, 6].

Disrupted sleep has emerged as both a symptom of and contributor to premature neurodegeneration. Short (≤ 6 hours) and long (≥ 10 hours) sleep durations, prolonged sleep latency, and reduced REM sleep have been linked to higher AD risk [7, 8]. These disturbances may act through pathways such as neuroinflammation, insulin resistance, melatonin dysregulation, and/or compromised blood–brain barrier integrity, which, in turn, accelerate cognitive decline [9–11]. Exercise appears to reduce neurodegeneration risk directly through neurogenesis and anti-inflammatory effects [12, 13] as well as indirectly through promoting improvements in sleep quality [14, 15]. Yet, few studies have disentangled the independent versus synergistic effects of sleep and exercise on cognition, AD-linked neuropathology, and cardiometabolic health in older adults who are at higher risk for AD.

Traditionally, neuropsychological testing often required clinical settings, which resulted in limited reachability and scalability. While recent advances in digital health technologies have resulted in the development of multiple remote, application-based cognitive assessments, there is an ongoing need for approaches that optimize the capturing of ecologically valid, real-world cognitive functioning, including cognitive function as it unfolds in naturalistic, dynamic, and contextually meaningful environments. This study will address this need by leveraging the Digital Neuro-Signature Platform, an augmented reality-based cognitive tool that simulates real-world tasks to assess global cognition as well as domain-specific cognitive function, such as learning and memory, executive function, and visuospatial skills [16, 17]. The Digital Neuro-Signature Platform is a novel contribution that builds upon the strengths of existing digital assessments by integrating immersive, behaviorally rich, and context-sensitive paradigms that may enhance sensitivity to subtle cognitive and functional changes that are relevant to AD prevention research. By pairing this with wearable technologies, including the Elemind Neuromodulation headband, Fitbit, and Lys light sensor, behavioral and physiological data on sleep and exercise will be unobtrusively captured while simultaneously improving assessment fidelity and participant engagement.

This pilot trial is designed to address critical scientific, clinical, and public health gaps by generating preliminary evidence at the intersection of sleep, exercise, and cognitive aging. Scientifically, the study will provide early-stage data on how two non-pharmaceutical, lifestyle-based health behaviors—sleep and physical activity—independently and jointly relate to cognitive performance, AD-linked neuropathology, and cardiometabolic risk in older adults with and without MCI. Clinically, the trial will evaluate the feasibility and signal of a novel, technology-enabled, scalable intervention across varying levels of cognitive risk, yielding effect size estimates and mechanistic insights to inform the design of larger, fully powered trials. Collectively, these findings will lay the groundwork for future personalized behavioral interventions and early-detection platforms that can be integrated into routine clinical and community-based care settings.

### Objectives

The main objectives of this exploratory, hypothesis-generating trial are to (1) assess whether improvements in sleep architecture (e.g., SWS, REM sleep), total sleep time, and sleep quality (e.g., increased sleep efficiency, decreased sleep onset latency and restlessness [restless bouts/hour]) are associated with improved cognitive performance and biomarker panels for brain and cardiometabolic health; (2) evaluate the effects of structured, moderate-level aerobic and anaerobic exercise on cognitive performance and biomarker panels for brain and cardiometabolic health; (3) examine the combined, potentially synergistic effects of the sleep and exercise interventions on cognitive performance and biomarker panels for brain and cardiometabolic health; and (4) investigate the role of light exposure as a potential modulator of sleep parameters.

The central hypotheses of this trial are that (1) disrupted baseline sleep architecture, short and long durations, and lower sleep quality measures as well as less physical activity will be linked with poorer cognition and more adverse brain and cardiometabolic biomarker profiles at baseline; (2) improvements in sleep metrics (i.e., increased sleep efficiency, SWS, REM, and total sleep time, as well as reduced onset latency and restlessness) will independently correspond to cognitive improvements and favorable biomarker changes during the 8-week intervention; (3) increased exercise will be independently associated with better cognitive, brain, and cardiometabolic health; (4) the combined sleep and exercise group will demonstrate greater improvements in cognitive function and biomarker profiles for brain and cardiometabolic health; and (5) these changes will be more pronounced in individuals with MCI compared to age- and sex-matched, cognitively normal (CN) controls. Given the short trial duration, this trial is explicitly intended for signal detection, building on similar 8-week studies of lifestyle-based behavioral interventions that have successfully detected early cognitive and physiological response patterns.

## Methods

### Study design overview

*Combating Alzheimer’s through Sleep and Exercise (CASE)* is a single-center, three-arm, randomized trial that will investigate the effects of sleep, exercise, and a combination of these on cognitive function as well as brain and cardiometabolic biomarker outcomes in older adults with and without MCI. Participants will be randomly assigned via Research Electronic Data Capture (REDCap [18, 19]) to one of three groups: (1) exercise-only, (2) sleep-only, or (3) combined sleep and exercise. Each group will receive the following intervention-specific digital tools: (1) all groups will receive a Fitbit to continuously track physical activity, including the tracking of all in-person, biweekly, 60-minute exercise interventions; (2) the sleep-only and sleep + exercise groups will receive the Elemind Neuromodulation headband for personalized acoustic stimulation; and (3) the sleep-only and sleep + exercise groups will receive a Lys device for monitoring light exposure.

The intervention will span 8 weeks and will follow structured phases, including screening, baseline assessment, intervention, and follow-up. Full Digital Neuro-Signature Platform augmented reality cognitive assessments will be used to assess cognition at baseline and week 8 (post-intervention) as well as biweekly as brief pulse assessments throughout the trial duration. Blood-based biomarkers will be collected via venipuncture at Quest Diagnostics at baseline and post-intervention under standardized fasting conditions. Specifically, in order to maximize participant safety and minimize any potential influences of acute exercise [20], the pre-intervention blood draws will be obtained no sooner than 24 hours and no later than seven (7) days before the initiation of the first exercise session, and post-intervention blood draws will be obtained within 24–48 hours after completion of the final exercise session. No blood draws will occur on the same day as in-lab exercise. Data from digital devices will be temporally synchronized using structured protocols to support integrated analysis, and raw sleep data will be reviewed for artifacts, with re-instruction provided for invalid nights. Patient-Reported Outcomes Measurement Information System (PROMIS) surveys [21] and validated physical activity questionnaires (including the Physical Activity Scale for the Elderly (PASE [22]) and the Community Healthy Activities Model Program for Seniors (CHAMPS [23])) will be administered via REDCap at baseline (week 0) and at week 8 during the in-person visits. The CASE study is designed to assess adherence, detect early efficacy signals, and explore behavioral mechanisms that may prevent or delay AD through modifiable lifestyle-based behavior interventions.

### Study procedures

#### Participants

Eligible participants must meet all of the following: (1) 55–80 years of age, reflecting growing evidence that individuals may experience increased AD risk starting in midlife [24, 25]; (2) able to safely perform moderate-intensity exercise as per the Physical Activity Readiness Questionnaire (PAR-Q, a validated exercise readiness questionnaire [26]); (3) able to wear and use Fitbit wristwatch and/or the Elemind Neuromodulation headband and Lys devices; (4) able to operate a digital device, such as mobile phone or computer; (5) able and willing to provide venous blood samples; (6) able to understand and speak English fluently (currently, there remains a lack of validated, multilingual versions of the Digital Neuro-Signature Platform and associated study interfaces; presently, such validated tools are only available in English, and the utilization of non-validated translations may introduce measurement bias, particularly regarding cognitive outcomes); (7) able to self-report a history of cognitive changes or impairment; and (8) be cognitively able (including those with MCI) and willing to provide informed consent.

Prospective participants will be excluded in the case of (1) severe mobility limitation(s) that prevent(s) safe participation in moderate exercise protocols; (2) unstable or uncontrolled medical conditions (e.g., recent cardiovascular event (≤ 6 months), cerebrovascular accident or transient ischemic attack (≤ 6 months), active cancer, or uncontrolled diabetes) that pose safety concerns; (3) inability to complete intake screening procedures; (4) severe uncorrected hearing impairment, particularly if it precludes effective utilization of the Elemind Neuromodulation headband, and/or vision impairment that interferes with digital device usage or in-person exercise guidance; (5) determined to have Alzheimer’s/dementia based on performance on the Quick Mild Cognitive Impairment (QMCI [27]; see Table 1); (6) inability or unwillingness to learn or engage with digital devices (e.g., smartphone, Fitbit, Elemind, or Lys), even with staff support; (7) regular pre-study engagement in ≥ 150 minutes of moderate-level and/or ≥ 75 minutes of vigorous-level exercise per week based on Fitbit data obtained during the initial 7-day onboarding screening period; (8) determined to have adequate sleep metrics, including duration (mean 7–9 hours) and quality (sleep efficiency ≥ 80% [28]), pre-intervention based on Elemind Neuromodulation headband data obtained during the initial 7-day onboarding screening period; and (9) current usage of disease-modifying therapy targeting amyloid (e.g., anti-amyloid monoclonal antibodies) or tau.

**Table 1:** Quick Mild Cognitive Impairment score thresholds by cognitive status

	**Aged ≤ 75, education < 12 years**	**Aged ≤ 75, education ≥ 12 years**	**Aged > 75, education < 12 years**	**Aged > 75, education ≥ 12 years**
CN	≥ 66	≥ 70	≥ 64	≥ 64
MCI	55–65	58–69	51–63	52–63
Dementia	< 55	< 58	< 50	< 51

Table describes the score thresholds of the Quick Mild Cognitive Impairment screening tool to suggest cognitive status (i.e., cognitively normal, mild cognitive impairment, or dementia) based on adjustments for dichotomized age and educational attainment. Higher scores indicate better cognitive function. *CN*, cognitively normal; *MCI*, mild cognitive impairment; *QMCI*, Quick Mild Cognitive Impairment screen

#### Participant recruitment

A total of 60 participants comprised of approximately 30 CN older adults and 30 older adults with MCI will be enrolled in this study. Recruitment strategies will consist of a combination of the following:

1. Participants will be recruited from existing studies and research registries maintained by the Translational Sleep and Circadian Sciences (TSCS) Laboratory at the University of Miami Miller School of Medicine. All individuals considered for enrollment will have previously consented to be contacted for future research opportunities. Initial contact will be made via phone by trained study personnel to confirm interest and screen for eligibility.
2. In addition, recruitment will include referrals for prospective participants with an MCI diagnosis from the Division of Cognitive Neurology at the University of Miami.
3. Moreover, flyers and social media advertisements will be distributed throughout southern Florida to further aid in recruitment efforts.
4. This trial will participate in the University of Miami’s Consent to Contact program, which allows approved researchers to access a database of University of Miami Health System patients who have agreed to be contacted about participating in clinical research studies.

Recruitment and enrollment procedures will comply with all institutional and federal guidelines, including the Health Insurance Portability and Accountability Act (HIPAA), to ensure privacy and confidentiality of all protected health information that is obtained. Eligible participants will be equally randomized (*n* = 20 per group) into three study arms: sleep-only, exercise-only, or combined sleep and exercise. Stratified randomization by cognitive status will be employed to ensure balanced representation across groups (i.e., each group will be comprised of 10 CN participants and 10 participants with MCI).

#### Screening and consent

A trained research assistant will conduct a brief phone pre-screening with all interested prospective participants. During this call, the research assistant will collect key demographic information (e.g., age, sex, education level, and smartphone ownership) and administer two validated screening tools: the Quick Mild Cognitive Impairment screen (QMCI, a clinician-administered tool used to assess objective cognitive status [27]) and the Physical Activity Readiness Questionnaire (PAR-Q, which evaluates the participant’s ability to safely engage in moderate exercise [26]; (see Table 2)). Both assessments will require fewer than five minutes to complete. Participants must achieve a QMCI score at or above the specified age- and education-adjusted scoring thresholds that indicate either CN or MCI status (see Table 1 [29, 30]) and report no contraindications to performing moderate-intensity exercise on the PAR-Q. Subjects who score below these thresholds will be excluded from the trial; no borderline adjudication process will be applied to ensure clarity and reproducibility.

**Table 2:** Screening questionnaires

Questionnaire	Description and variables collected
Basic Info Screening Form (eligibility criteria)	Age, whether prospective subjects own and can operate a smartphone/computer device
Physical Activity Readiness Questionnaire (PAR-Q)	Safe for participants to perform moderate-level exercise. This will be the main factor in determining if it is safe to perform the moderate-intensity exercise routine for participants; an emergency plan is detailed later in the study
Quick Mild Cognitive Impairment screen (QMCI) 6-item	A clinician-administered tool used to assess objective cognitive status

Table describes the screening questionnaires that will be to evaluate the eligibility of each prospective participant as well as a descriptive identification of which variables of particular interest will be collected

Individuals meeting these pre-screening criteria will then be scheduled for an in-person screening visit to provide written informed consent and further assess eligibility criteria via a 7-day screening period. After signatures are obtained, each prospective subject will be provided with a copy of the signed informed consent document. All procedures have been approved by the University of Miami Institutional Review Board (IRB) requirements and federal privacy protections and will be conducted in accordance with the Helsinki Declaration. Thereafter, prospective participants will be randomized into one of three arms and will receive intervention-specific, wearable devices and will be instructed on how to use these devices. All prospective participants will receive Fitbit devices, while those assigned to sleep or sleep + exercise intervention will receive an Elemind Neuromodulation headband to be used daily without stimulation during the initial 7-day screening period and a Lys device. Prospective participants in all groups will be asked to wear the Fitbit device continuously to establish baseline physical activity levels and to confirm sedentary to lightly active physical activity levels in order to satisfy eligibility criteria for those assigned to interventions that involve exercise, and those assigned to the sleep-only or sleep + exercise interventions will be asked to wear the Elemind Neuromodulation headband without stimulation while the device actively collects sleep data for seven consecutive nights to establish baseline sleep patterns, ensure comprehension of and comfortability with wearing the headband; additionally, this will confirm subquality sleep pre-intervention to satisfy eligibility criteria for those assigned to groups that involve the sleep intervention.

Prospective participants who are assigned to the sleep or sleep + exercise interventions and are determined to have good quality sleep after the onboarding period as defined by the eligibility criteria will either be reassigned to the exercise-only intervention (if also found to engage in < 150 minutes of moderate-level or < 75 minutes of vigorous-level activity weekly via the Fitbit) or will be excluded from further participation and given $20 compensation. Prospective participants who are assigned to the exercise or sleep + exercise interventions and who are found to be active or very active (as defined by the eligibility criteria) will either be reassigned to the sleep-only intervention and be invited to undergo an additional 7-day onboarding screening duration with the Elemind Neuromodulation headband to determine sleep-specific eligibility or will be excluded from further participation and given $20 compensation. Prospective subjects who agree to undergo an additional 7-day screening period and are deemed to have good quality sleep pre-intervention at baseline will also be excluded from participation and given $20 compensation.

Once a participant has completed the 7-day onboarding screening period and found to be eligible, subjects will be prompted to set up an appointment with Quest Diagnostics. Sample analysis will be available to the participant through email and/or through the Quest Diagnostics app within a few days, and participants will consent to share the data with the University of Miami study team. Intervention (week 0) will begin as soon as possible after this process is complete. Another identical procedure will be initiated as soon as possible after the intervention period is complete. All procedures will comply with the University of Miami Institutional Review Board (IRB) requirements and federal privacy protections. Subsequent to completing the onboarding screening period, eligible participants will be invited to complete an in-person baseline visit.

#### Randomization

The design will follow CONSORT randomization guidelines [31]. Participants will be eligible for randomization once all baseline questionnaires are completed. Blocked randomization will be conducted using a randomization table. Subjects in each block will be assigned to one of three intervention arms, ensuring equal allocation between CN and MCI subjects in each group.

#### Baseline and follow-up visits

Data will be collected at baseline, biweekly, and at the 8-week follow-up to assess changes in sleep, exercise, biomarkers, and cognition. After consent and randomization, participants will be onboarded in person to each intervention-specific device and observed for a 7-day period prior to the baseline visit to establish baseline sleep patterns and/or physical activity levels relevant for each assigned intervention and to ensure all eligibility criteria are satisfied. Cognitive function will be measured using an augmented reality-based tool at baseline and week 8, with brief pulse assessments administered twice-weekly throughout the intervening period. Blood biomarkers will be collected via venipuncture at Quest Diagnostics (baseline and week 8). Physical activity and sleep data will be respectively monitored using Fitbit (all groups) and the Elemind headband (sleep and sleep + exercise groups), and light exposure will be tracked via the Lys device (sleep and sleep + exercise groups). Participants will also complete validated self-report surveys (e.g., PASE, CHAMPS–MET, PROMIS instruments) to capture psychosocial and behavioral variables at weeks 0 and 8. This multimodal approach will enable temporally-aligned, comprehensive data capture across digital and self-reported domains (see Table 3).

**Table 3:** Timeline of questionnaires provided

Variable	Screening	Baseline	8-week
Basic Info Screening Form (eligibility criteria)	X		
Physical Activity Readiness Questionnaire (PAR-Q)	X		
Quick Mild Cognitive Impairment screen (QMCI), 6-item	X		
Demographic Questionnaire		X	
Chronic Health Questionnaire		X	X
PROMIS Self-reported Cognitive Functioning Behaviors Short Form		X	X
PROMIS Sleep Disturbance 4a		X	X
PROMIS Emotional Support 4a short form		X	X
PROMIS Depression 4a shot form		X	X
PROMIS Anxiety 4a short form		X	X
PROMIS Social Isolation 4a		X	X
General PROMIS Life Satisfaction 5a		X	
Cross Ethnic-Racial Identity Scale		X	
PASE Physical Activity Questionnaire (exercise and sleep + exercise interventions only)		X	X
CHAMPS-MET Physical Activity Questionnaire (exercise and sleep + exercise interventions only)		X	X
**Biomarker data**			
Total cholesterol		X	X
High density lipoproteins		X	X
Low density lipoproteins		X	X
Triglycerides		X	X
High-sensitivity C-reactive protein		X	X
Glycated hemoglobin		X	X
C-peptide		X	X
Insulin		X	X
Neurofilament light chain		X	X
Amyloid beta peptide 1–42		X	
Amyloid beta peptide 1–40		X	
Amyloid beta peptide 1–42/amyloid beta peptide 1–40 ratio		X	
Phosphorylated tau at threonine 217		X	

Table outlines the timeline for the collection of information from questionnaires and blood panels. *PROMIS*, Patient-Reported Outcomes Measurement Information System.

Participants who provided written consent and are found to be eligible for participation at the conclusion of the 7-day onboarding screening period will undergo the following procedures in accordance with the intervention to which each subject was assigned:

### Interventional group 1 (exercise only)

Participants randomized to the exercise-only group will engage in an 8-week, home- and site-based exercise intervention designed for older adults across a range of cognitive and physical function levels. All site-based sessions will occur in-person at the study facility under supervision of trained staff. Each week, participants will attend two 60-minute structured exercise sessions, consisting of aerobic training and resistance training. On the remaining five days when subjects will not engage in structured exercise, subjects will be asked to engage in 30 minutes of moderate-intensity walking activities. In-lab exercise protocols will be fully standardized to ensure consistency of exercise dosage and fidelity across participants. The target exercise load will be 270 minutes of moderate-intensity exercise weekly for 8 weeks. Although considered short-term exercise, this exercise dosage has been shown to improve cerebral blood flow, regional brain volume, and brain-protective trophic factors [32].

Participants will begin with a 7-day onboarding period, during which participants will wear the Fitbit continuously to establish baseline physical activity levels and to ensure initial eligibility criteria are satisfied. Subsequent to this initial observation and screening phase, subjects will participate in biweekly, in-person exercise sessions, which will be comprised of 60 minutes of stretching, aerobic, and resistance training exercises. Specifically, exercise sessions will consist of a 10-minute warm-up, followed by 20 minutes of moderate-intensity aerobic exercise at 60–75% of estimated maximum heart rate (MHR). MHR will be estimated using Tanaka’s formula (maxHR = 208-(0.7*age)) [33], and the target heart rate (THR) will be calculated using Karvonen’s formula to account for resting heart rate (RHR), where THR = ((MHR-RHR)*% intensity) + RHR [34]. Moderate-intensity aerobic training will be followed by 20 minutes of resistance training of major muscle groups with an emphasis on lower extremity resistance training one day each week and upper extremity resistance training at the second in-person session weekly. Exercise load will be at 60–80% of an estimated one repetition max using American College of Sports Medicine Guidelines [35]. Muscle groups that will be trained include biceps, triceps, deltoid, and latissimus dorsi for the upper extremity, and quadriceps, hamstrings, and gastrocnemius/soleus for the lower extremity. Resistance training exercises targeting the trunk will also be incorporated. Each exercise session will then conclude with 10 minutes of post-exercise stretching.

#### Home-based protocol

In addition to the twice-weekly, site-based exercise sessions, participants will be asked to include 30 minutes of walking at moderate intensity on the remaining five days each week when subjects do not complete the biweekly, in-person exercise sessions.

#### Assessment of aerobic capacity

Aerobic capacity will be measured at baseline and after 8 weeks of exercise using the six-minute walk test (6MWT). The 6MWT accurately estimates maximal oxygen uptake capacity (VO2max) using regression equations, including distance walked, sex, age, and body mass index [36].

#### Cognitive pulse assessments

Participants will complete twice-weekly, brief cognitive pulse assessments using a digital cognitive tool, which will take approximately 10–20 minutes to complete. These will occur immediately after each in-lab exercise session in a designated testing space, enabling the study team to capture near-term changes in cognitive function related to exercise. Additionally, participants will complete the full-length digital cognitive assessments at week 0 (baseline) and week 8 in a quiet testing room. These assessments will simulate real-world cognitive tasks and will yield high-resolution, domain-specific cognitive profiles.

#### Continuous physical activity monitoring

Participants will wear a Fitbit continuously throughout the study, including during both wake and sleep hours. Fitbit-derived data will be categorized into three activity domains:

1. Protocol-Guided Exercise (PGE): Exercise occurring during supervised, in-lab sessions.
2. Non-Protocol Physical Activity (NPA): Incidental movement and unsupervised activity outside scheduled sessions.
3. Total Physical Activity: Combined PGE + NPA, derived from 24-h Fitbit data.

The primary Fitbit metric will be daily step count, a validated proxy for global movement shown to correlate with cognitive, brain, and cardiometabolic health outcomes in older adults [37]. A secondary metric, calories burned, will offer insight into the metabolic intensity of movement. Step count and caloric expenditure will be examined in relation to cognitive function as well as brain and cardiometabolic biomarker outcomes. Analytical models will include PGE and NPA as independent predictors, which will be modeled separately and jointly to assess differential and additive contributions to primary (cognitive, brain, and cardiometabolic markers), secondary (sleep metrics and exercise measures), and tertiary (light exposure and psychosocial measures) outcomes.

#### Self-reported physical activity

To complement wearable-derived data and assess convergent validity between objective and subjective measures of physical activity, participants will complete two validated, self-report instruments. Specifically, the Physical Activity Scale for the Elderly (PASE; [22]) will be administered at week 0 (baseline) and week 8 (post-intervention). This questionnaire will capture the frequency, duration, and intensity of physical activity over the prior 7 days. In addition, participants will complete the Community Healthy Activities Model Program for Seniors (CHAMPS) questionnaire, which evaluates the effectiveness of an exercise intervention in community-dwelling older adults. From the CHAMPS questionnaire, a Metabolic Equivalent of Task (CHAMPS–MET) metric will be derived; this metric will be utilized to estimate weekly caloric expenditure [23]. The CHAMPS survey will also be administered at week 0 (baseline) and week 8 (post-intervention). The integration of these two tools will enable a comprehensive triangulation strategy to optimally evaluate alignment and potential discrepancies between perceived and actual physical activity. Discrepancy scores between self-reported and Fitbit data will be computed and examined as potential moderators or mediators of outcomes, particularly in relation to cognitive status and/or adherence.

#### Biomarker collection strategy

Brain and cardiometabolic health will be assessed using a hybrid biospecimen collection protocol. Two venipuncture-based blood draws will be conducted through Quest Diagnostics, occurring at week 0 (pre-intervention) and week 8 (post-intervention) under standardized fasting conditions. To maximize participant safety and minimize all possible influences of acute exercise [20], pre-intervention blood draws will be obtained no later than 7 days prior to, but no sooner than 24 hours before, the initiation of the first exercise session, and post-intervention blood draws will be obtained within 24–48 hours after completion of the final exercise session. No blood draws will occur on the same day as in-lab exercise. For all participants, plasma samples will be assessed for cardiometabolic risk, which will include high-sensitivity C-reactive protein (hs-CRP), glycated hemoglobin (HbA1c), and fasting total cholesterol, high-density lipoproteins (HDL), low-density lipoproteins (LDL), triglycerides, insulin, and C-peptide levels, which will provide time-sensitive insight into the inflammatory, glycemic, lipid, and insulin resistance markers across the intervention period. Furthermore, the extent of AD-associated neuropathology will be assessed at baseline via plasma samples, which will include amyloid beta peptide 1–42 (Aß1–42), Aß1–40, Aß1–42/Aß1–40 ratio, and phosphorylated tau at threonine 217 (p-tau217). These biomarkers will be subsequently integrated into a single analytical interpretation of AD risk, which has an 88% positive and 91% negative concordance with amyloid-PET-based results [38]. The aforementioned biomarkers will serve as staging and stratification variables, enabling the utilization of such biomarkers as covariates or effect moderators (e.g., assessing whether intervention response differs by baseline pathology status) without presuming measurable longitudinal change over the short intervention window. Additionally, in order to capture biologically meaningful change over an 8-week duration, plasma neurofilament light chain (NfL) will be obtained and measured at baseline and at post-intervention. Unlike the amyloid- or tau-based biomarkers, NfL reflects ongoing neuroaxonal stress and overall neurodegeneration and has demonstrated sensitivity to behavioral and lifestyle interventions, including exercise- and sleep-related alterations, over relatively short time periods (e.g., 8 weeks) [39]. Consequently, NfL is more appropriate for detecting intervention-related biological signals within the timeframe of this pilot study.

#### Temporal synchronization and data integration

Physical activity, cognitive function, and biomarker data will be aligned using a centralized time-stamped database. Sampling windows will be synchronized with protocol events (e.g., in-lab sessions, assessment points) to ensure precise temporal integration of physiological, cognitive, and behavioral data streams.

#### Adherence and engagement

In order to assess adherence to the exercise sessions, attendance will be recorded for all in-person sessions, and wearable devices (Fitbit) will track daily physical activity, including steps, heart rate, and durations of physical activity. Weekly check-ins (10–15 minutes) via phone or secure teleconference will be conducted and documented to ensure protocol adherence, troubleshoot technical issues, and maintain engagement. All data will be stored in HIPAA-compliant systems. Fitbit and the Digital Neuro-Signature Platform data will be de-identified and linked using secure participant codes. Statistical models will include age, sex, years of educational attainment, employment status (unemployed/employed), socioeconomic status, baseline sleep metrics, baseline physical activity levels, and cognitive status as covariates. By integrating standardized, in-lab exercises, wearable technology, digital cognitive assessments, and multimodal biomarker data, this arm of the *CASE* study aims to explore the cognitive, brain, and cardiometabolic impacts induced by structured exercise in aging adults who are at higher risk for AD using a real-world, ecologically valid framework.

### Interventional group 2 (sleep only)

**Fig. 1:**
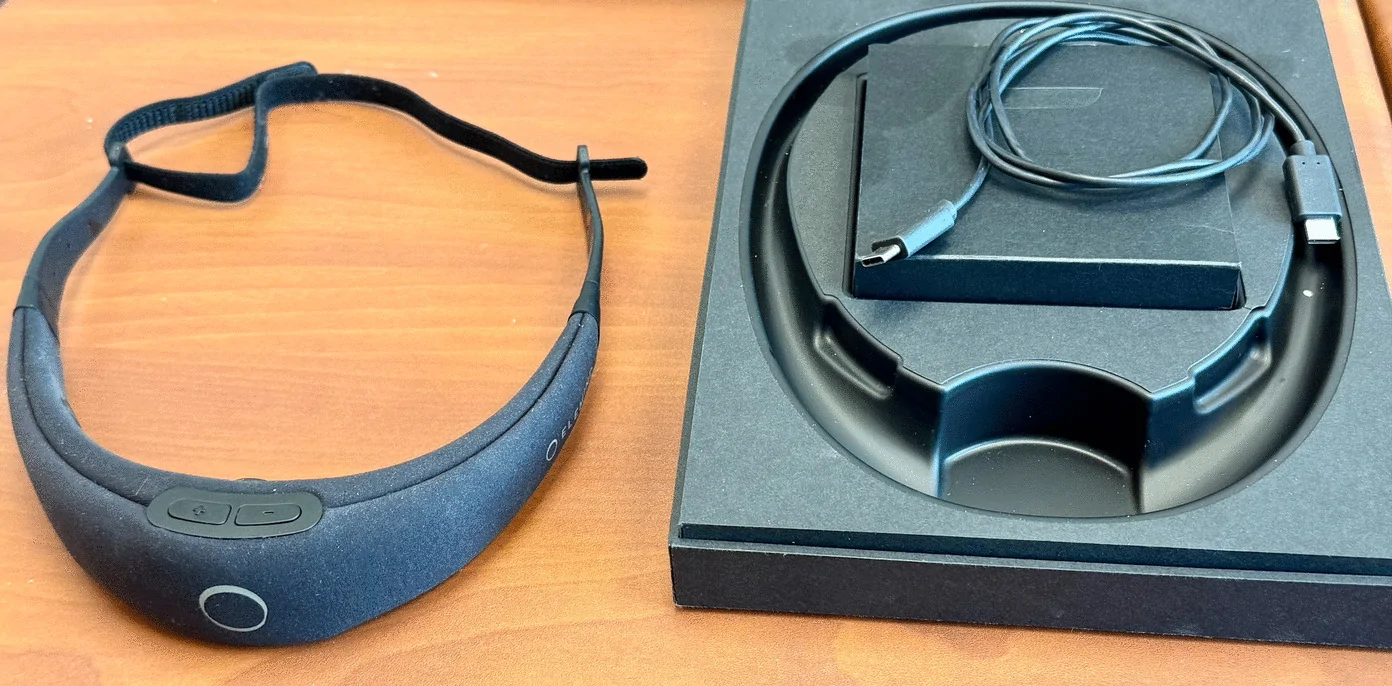
Elemind Neuromodulation Headband. The wearable Elemind Neuromodulation headband (left) promotes a reduction in sleep onset latency through acoustic stimulation based on each individual’s unique brainwaves (left). The Elemind Neuromodulation headband can be utilized in conjunction with machine learning approaches to optimize personalized treatments. The user-programmed device will use Bluetooth low energy or a direct USB-C connection (right) to control the device and transfer data obtained by the headband to the Elemind application on a smartphone, which will collect sleep metrics

Participants randomized to the sleep-only group will participate in an 8-week intervention utilizing the Elemind Neuromodulation headband, an electroencephalography-based, non-invasive, wearable device designed to reduce onset latency through personalized acoustic stimulation that is tailored to the unique brainwaves of each individual (see Fig. 1). This arm is intended to assess the efficacy of a home-based neuromodulation strategy in older adults with varying cognitive and functional baselines.

#### Device use and sleep monitoring

Subjects will start with a 7-day onboarding-screening period, during which participants will wear the Elemind Neuromodulation headband nightly without stimulation to passively establish a baseline sleep pattern and to ensure initial eligibility criteria are satisfied. Following this, the neuromodulation feature will be activated, and participants will be instructed to wear the device at least 3–4 nights per week for 8 weeks. Each use will deliver bone-conduction-based acoustic stimulation tailored to the participant’s real-time brainwave activity. The headband will collect continuous data on multiple sleep metrics, including total sleep time, sleep efficiency, sleep onset latency, restlessness, and time spent in various sleep stages (light (N1/N2), N3, REM). Sleep data will be delivered to the Elemind smartphone application. Raw data will be reviewed regularly for artifacts, such as signal dropout and excessive motion. Nights with incomplete data (e.g., < 4 hours of recording or ≥ 10% missing epochs) will be flagged and excluded from analysis and will prompt follow-up instructions and/or re-onboarding support. The Elemind Neuromodulation device’s sleep staging outputs, while designed to approximate polysomnographic benchmarks, are considered proxies and will be interpreted accordingly. In addition, participants will wear the Lys device continuously throughout the 8-week trial period. The Lys device will measure the intensity, color, and duration of light exposure to gain insights into the relationship between light, sleep, circadian cycles, and energy levels. This device works by communicating with the company’s proprietary app, named Insight, to display light exposure data to the user as well as to provide the individual with goals and insights related to light and health. Additionally, Lys communicates with a separate app, called Collect, which will be used by the researchers to download data at the conclusion of the study. In addition, subjects in this group will also receive Fitbit devices and will be asked to wear the device continuously to passively gauge physical activity levels throughout the trial duration.

#### Cognitive pulse assessments

Full-length, digital cognitive assessments (approximately 20 minutes) will be conducted at week 0 and week 8 in a quiet testing room at the University of Miami or, if unable to complete in person, via a secure video conference with a trained research assistant or postdoctoral fellow. Throughout the study duration, participants in this group will also complete brief, twice-weekly cognitive pulse assessments in each participant's  own home setting using the Digital Neuro-Signature Platform cognitive tool on two mornings per week after wearing the Elemind Neuromodulation headband the night before.

#### Biomarker collection strategy

Similar to the first arm, cardiometabolic and brain health will also be assessed using a hybrid biospecimen collection protocol in this arm. Specifically, two venipuncture-based blood draws will be conducted through Quest Diagnostics, occurring during week 0 (pre-intervention) and week 8 (post-intervention) after the final utilization of the Elemind Neuromodulation headband has been completed. Samples will be assessed for hs-CRP and HbA1c levels as well as fasting total cholesterol, HDL, LDL, triglycerides, insulin, and C-peptide levels, which will provide time-sensitive insight into the inflammatory, glycemic, lipid, and insulin resistance markers across the intervention period. Changes in neuroaxonal stress and overall neurodegeneration from baseline will be assessed via plasma NfL levels at weeks 0 and 8. Furthermore, AD-linked neuropathology will be assessed at baseline only via plasma samples, including Aß1–42, Aß1–40, Aß1–42/Aß1–40 ratio, and p-tau217.

#### Missing data and adherence

Participants missing ≥ 10% of required sleep intervention nights (defined as < 22 nights of wearing the sleep intervention over the 8-week intervention period) will be flagged for sensitivity analyses. If a participant misses > 2 consecutive weeks of data and/or fails to complete either of the full-length, digital cognitive assessments, such participants will be excluded from per-protocol analyses but retained in intention-to-treat models. Multiple imputations will not be applied due to the small sample size; instead, pattern mixture modeling or last observation carried forward (LOCF) methods will be used where applicable.

Adherence to the recommended neuromodulation utilization for the sleep intervention will be monitored via the Elemind application, which will log device usage and track sleep metrics. In addition, weekly phone or video check-ins (10–15 minutes) will be made to monitor engagement, support adherence, and troubleshoot technical issues. All reports of adherence will be documented upon the completion of each call/video check-in.

#### Limitations

This study will not include a sham stimulation control for the sleep-only group. The decision to forgo a sham condition reflects the real-world, comparative effectiveness design of the CASE study and prioritizes ecological validity over internal blinding. Nonetheless, the absence of participant blinding may introduce potential placebo effects. To address this analytically, expectancy-related influences will be modeled using self-reported perceived benefit measures as an additional covariate in the regression models. Sensitivity analyses will explore whether high expectancy ratings moderate intervention effects. Between-arm comparisons will further contextualize the potential role of non-specific treatment effects in observed outcomes.

In addition, the 8-week interventional period of this clinical trial may not be a sufficient timeframe for detecting significant changes in brain biomarkers; nonetheless, it should be emphasized that the predominant goal of this pilot trial is to identify early trends and signals in these aforestated biomarkers, which will, in turn, inform the design of future, longer-term, fully powered trials. Furthermore, this exploratory approach will enable the assessment of the feasibility and potential effects of each intervention in a relatively small cohort and prefaces more extensive future studies. Notably, the 8-week interventional trial duration was selected based on prior study findings, which showed that behavioral interventions have the capacity to result in early changes in cognitive and physiological measures within a relatively short timeframe [40, 41].

#### Temporal synchronization

All data sources—including Elemind (sleep), Lys (light exposure), Fitbit (physical activity/exercise), and digital cognitive assessments (cognitive function)—will be time-stamped and aligned using a centralized database. Sampling frames will be linked to specific intervention events and assessment windows to enable a temporally precise integration of cognitive and sleep data streams. By integrating wearable neurotechnology, frequent cognitive assessments, and remote monitoring within a structured intervention framework, this arm of the CASE study aims to evaluate the adherence and early efficacy signals of at-home sleep neuromodulation in aging adults.

### Interventional group 3 (combined sleep and exercise)

Participants randomized to the combined intervention group will engage in both structured exercise sessions and sleep neuromodulation over an 8-week study period. This arm is designed to explore the additive and potentially synergistic effects of engaging in both interventions concurrently while assessing adherence and participant burden, which will, in turn, inform future multimodal approaches to cognitive aging and AD prevention.

#### Protocol structure and considerations

To reduce participant burden and improve standardization, in-lab exercise sessions and cognitive assessments will follow the same cadence and structure as in the exercise-only arm: twice-weekly, 60-minute, supervised sessions that will focus on aerobic and resistance training, home-based walking program, and brief digital cognitive assessments (the Digital Neuro-Signature Platform) immediately after each session. Participants will also wear the Elemind Neuromodulation headband for at least 3–4 nights per week across the 8-week period, with an initial 7-day, passive, onboarding screening phase of wearing the headband nightly without stimulation in combination with wearing the Fitbit device continuously to establish baseline sleep patterns, physical activity levels, and ensure that all eligibility criteria are satisfied. Finally, subjects will also wear the Lys device continuously throughout the 8-week trial period.

To manage cumulative burden, scheduling flexibility will be emphasized. Although participants will be offered a choice of days and times for in-lab sessions to fit each participant’s weekly routines, morning times will be strongly encouraged across all participants, as prior work has suggested that older adults generally show better cognitive and exercise performance in the mornings [42, 43]; this will further improve standardization. Staff will work closely with participants to accommodate visit times and ensure ample time for recovery between activities. Weekly adherence and well-being check-ins will be conducted and documented to monitor fatigue, motivation, and adverse effects.

#### Cognitive pulse assessments

As in the other arms, participants will complete brief, digital cognitive assessments biweekly during lab visits and full-length assessments at week 0 and week 8. For improved standardization, participants in this group will also be asked to attend in-person exercise sessions on the days after they wore the Elemind Neuromodulation headband the preceding night.

#### Biomarker collection strategy

Biomarker collection through Quest Diagnostics (venipuncture at week 0 and week 8) will be conducted following the aforementioned procedures, with attention to minimizing scheduling conflicts with intervention activities. Just as in the exercise-only intervention, pre-intervention blood draws for this arm will occur no sooner than 24 hours before but no later than 7 days prior to commencing with the initial exercise session. Additionally, post-intervention blood draws will be obtained within 24–48 hours after completion of the final exercise session. No blood draws will occur on the same day as a scheduled exercise session.

#### Analysis strategy

This arm enables a unique opportunity to assess both the independent and combined contributions of sleep and exercise to cognitive, brain, and cardiometabolic outcomes. Statistical models will include main effects for sleep (e.g., adherence to Elemind usage), exercise (e.g., PGE, NPA), and interaction terms to evaluate additive or synergistic effects. Dose-response relationships will be explored using adherence metrics and physiological markers (e.g., heart rate during exercise, sleep efficiency). Adherence to both modalities will be assessed as previously described, summarized descriptively, and modeled as predictors in multivariable analyses of primary outcomes.

#### Compensation and parking reimbursement

All participants will receive $50 at baseline (week 0) and $50 at the conclusion (week 8) visit, totaling up to $100 for full participation in the trial. Participants will be issued a reloadable debit card (ClinCard) that can be used for study-based compensation at the University of Miami. Funds will be loaded onto the card according to the payment schedule. The study staff will provide information about how the card works. The company administering the card will require a full name, address, and date of birth to be entered online for payment. This information will only be used for payment and communication purposes and will not be given to another company or linked to any of the study data. All prospective participants who pass pre-screening eligibility but are found to be ineligible after the initial 7-day onboarding screening period and are also not eligible for or interested in being reassigned to an alternative intervention group for which they may be eligible will not be invited for further participation but will receive $20 compensation for their efforts via a ClinCard debit card. Furthermore, for all in-person visits, participants will be reimbursed for either parking or via Metro passes at the conclusion of each in-person visit.

### Measures and instruments

To capture a comprehensive and multidimensional view of participants’ cognitive, emotional, behavioral, and physiological profiles, the study will employ both survey-based instruments and digital device-based measures. Instruments will be categorized into predictor and outcome variables and will span psychosocial, cognitive, behavioral, and biological domains.

Self-report surveys will be administered at baseline (week 0) and at the 8-week follow-up by a trained research assistant or postdoctoral scholar via in-person visits or, if unable to come in person in the sleep-only group, by a secure video conference. These will include the PASE [22] and CHAMPS [23] questionnaires to assess physical activity and weekly caloric expenditure, respectively, for those in the exercise-only or sleep + exercise interventions. Additionally, each participant will complete all validated Patient-Reported Outcomes Measurement Information System (PROMIS) short-form questionnaires that measure sleep disturbance, self-reported cognitive functioning, emotional support, depression, anxiety, life satisfaction, and social isolation [21] at the baseline and post-intervention visits, except for the General PROMIS Life Satisfaction 5a questionnaire, which will be completed at the baseline visit only. Additional measures will include a Demographic Questionnaire, a Chronic Health Questionnaire, and the Cross Ethnic-Racial Identity Scale [44] to capture relevant sociodemographic and psychosocial predictors (see Table 3). Objective data will be collected via digital devices, including the Elemind Neuromodulation headband (sleep metrics), Fitbit Charge 5 (physical activity and heart rate), Lys sensor (light exposure), and the Digital Neuro-Signatures neuropsychological assessment (cognitive function). Blood-based biomarkers will be obtained through Quest Diagnostics to assess brain and cardiometabolic health for all participants at the baseline and post-intervention visits as well as AD-associated neuropathology for all participants at the baseline visit. The 6MWT will be used to assess changes in fitness and aerobic capacity. Together, these tools will enable high-resolution modeling of the relationships between behavior, biology, and cognitive health across all three study arms.

#### Physical Activity Scale for the Elderly (PASE) questionnaire

The PASE Questionnaire will only be administered to participants assigned to interventions involving exercise. The PASE is a validated, self-report instrument that is widely used to assess physical activity. The PASE Questionnaire was originally designated for community-dwelling adults aged ≥ 65 years but has been subsequently validated in adults aged 45–64 years [45]; this questionnaire has demonstrated high test-retest reliability, with intraclass correlation coefficients (ICC) found to be *r* = 0.75 (95% CI = 0.69–0.80) over 3–7 weeks [22]. Short-interval reliability (10–14 days) has shown excellent reproducibility with ICCs, which range from 0.93–0.99 in prior work [46, 47]. Furthermore, construct validity is moderately supported: correlations with accelerometer-measured physical activity (steps, energy expenditure) range from *r* ≈ 0.30–0.61 [48]. While internal consistency varies across activity domains (α ≈ 0.69–0.99 [22, 46]), the total score remains robust for classifying overall activity levels in older populations. Given the robustness of the PASE questionnaire across older adult populations, similar reliability is expected in upper middle-aged adults (55–64 years).

#### Community Healthy Activities Model Program for Seniors (CHAMPS) questionnaire

Similarly to the PASE questionnaire, the CHAMPS questionnaire will also be administered only to participants assigned to interventions involving exercise. The CHAMPS questionnaire is a validated, self-reported instrument that was originally designed to evaluate the effectiveness of an exercise intervention in community-dwelling older adults aged ≥ 65 years but has subsequently been demonstrated to be a valid tool to utilize for middle-aged participants as well [49, 50]. From the CHAMPS questionnaire, a Metabolic Equivalent of Task (CHAMPS–MET) metric can be derived. The CHAMPS-MET metric will be utilized to estimate weekly caloric expenditure [23]. The CHAMPS questionnaire has demonstrated acceptable construct validity and reliability in older adult cohorts, with reported correlations of *r* ≈ 0.23–0.55 when compared with accelerometer-based measures or functional health indicators [23, 49]. The CHAMPS questionnaire effectively captures activities typical of older adults’ daily routines and complements objective, wearable metrics.

#### Fitbit charge 5 wearable metrics

Consumer-grade Fitbit devices, including the Charge series, have demonstrated acceptable validity and reliability for measuring steps and energy expenditure in older adults. In controlled laboratory settings, Fitbit-measured steps agreed within ± 3% of observed counts; in free-living conditions, deviations remained within approximately ± 10% of research-grade accelerometers (e.g., actigraphy [51]). Prior evidence indicates that step counts correlate well with self-reported metrics—specifically, Fitbit steps with the PASE questionnaire and Fitbit calories burned with the CHAMPS–MET metric, particularly in older adult populations [37]. While Fitbit may underestimate steps at slower gait speeds [52], overall metrics are sufficiently reliable for longitudinal monitoring in aging cohorts. Data collection from this device will consist of two steps: (1) a period of data collection while participants initially wear the Fitbit for seven consecutive days before the intervention is implemented and (2) subsequent data collection while participants continue wearing the Fitbit throughout the intervening period. Similarly to the interventions that include exercise, participants in the sleep-only group will also be asked to continuously wear the device throughout the 8-week trial duration to ensure that physical activity levels do not confound the effects of sleep.

It should be noted that there is a considerable paucity of evidence in the prevailing body of literature assessing how well Fitbit-derived metrics correlate with PASE and CHAMPS-MET scores in upper middle-aged adults (i.e., 55–64 years). As a result of this age-specific validation gap, the current trial will combine Fitbit metrics (e.g., step counts and caloric expenditure) with self-reported physical activity data from the PASE and CHAMPS questionnaires; this approach will enable a more comprehensive assessment of physical activity patterns and provide an opportunity to compare and interpret agreement between objective and self-reported measures within this cohort. The paucity of evidence that has assessed these correlations with the inclusion of middle-aged and older adult samples has generally found moderate relationships between Fitbit-derived step counts and PASE scores as well as between Fitbit-derived estimated energy expenditure and CHAMPS-derived MET values [37].

#### Elemind Neuromodulation headband

The Elemind Neuromodulation headband is a wearable device that utilizes noninvasive, electroencephalography-based (EEG-based) technology to induce a sleep state more rapidly via personalized acoustic stimulation (see Fig. 1). Specifically, the headband uses a closed-loop system, meaning that the headband conveys stimulation based upon recorded neural activity in order to modulate real-time oscillating circuits at the macroscopic level [53]. Technologies that utilize closed-loop systems have been shown to be a superior approach to open-loop systems for disease treatment in prior work [54]. This device uses five dry, flexible EEG electrodes—including three recording electrodes that are positioned at the Fpz and Fp1/2 electrode sites in accordance with the 10–20 International System of electrode placement and two silicone rubber-based, linked reference electrodes located at the mastoids—to measure neural activity during sleep and provide an estimation of the phases of real-time neural oscillations [55, 56].

The headband generates a sound pulse by identifying alpha neural oscillations in real-time through endpoint-corrected Hilbert Transform algorithm, a signal processing advancement that is independent of oscillation sources and utilizes an infinite impulse response causal bandpass filter [53]. The headband furthermore utilizes a bone-conduction driver built into the center of the headband, which anatomically corresponds to the central forehead, to deliver auditory stimulation that is locked to phases of brainwave activity [55, 56]; this strategy has been found to impact continuous cortical oscillatory activity in prior studies [57, 58], including in older adults [57]. This method has, in turn, been shown to improve sleep for not only SWS oscillations [55, 57, 59] but also for oscillations across all sleep stages [56].

The Elemind Neuromodulation headband can be utilized in conjunction with machine learning approaches to optimize personalized treatments [53]. As such, the user-programmed device will use Bluetooth low energy or a direct USB-C connection to control the device and transfer data obtained by the headband to the Elemind application on a smartphone [55, 56], which will collect sleep metrics, including: (1) total sleep time, (2) sleep efficiency, (3) sleep architecture durations and percentage of total sleep time spent in each sleep stage (i.e., durations and percentage of total sleep time spent in the light (N1/N2), SWS, and REM stages of sleep); (4) sleep onset latency; and (5) restlessness (number of restless bout(s) per hour). The headband device will be supplied to participants at baseline and collected at the end of the study to analyze the sleep data. This intervention process will consist of two steps: (1) a period of data collection while participants initially wear the headband without stimulation for seven consecutive days and (2) subsequent data collection while participants continue wearing the Elemind Neuromodulation headband intervention with stimulation for at least 3 to 4 days per week throughout the 8-week duration. At the end of the analysis phase, the Elemind app will deliver a sleep report that includes a summary of each week’s aforementioned sleep data while the headband was in use. As each participant must have their own unique accounts to access the data associated with the utilization of the Elemind Neuromodulation device, there will be no data breach risk between participants who use the same Elemind device after each participant has completed the trial.

#### Lys

Lys is a coin-sized device which tracks lux and color levels through sensors aiming to replicate the eye’s photoreceptors. This device clips onto clothing or close-contact accessories (e.g., purse strap) and tracks light information throughout the day. The Lys devices measure the intensity, color, and duration of light exposure to gain insight into the relationship between light, sleep, circadian cycles, and energy levels. The Lys device communicates with the company’s proprietary app, named Insight, to display light exposure data to the user, as well as to provide the user with goals and insights related to light and health. The Lys device also communicates with a separate app, called Collect, which is used by the study team to download all data at the conclusion of the study. The Lys device has been validated in previous literature and has been used in other studies of this nature [60].

#### Quest Diagnostics blood draw

Participants will go to the nearest Quest Diagnostics location for blood sample collection. This de-identified data collected by Quest (i.e., biomarker data linked to a participant by a study identification number) will be consented to be shared by the participant to the study team. Cardiometabolic biomarkers will be collected and analyzed for all participants at the baseline and post-intervention visits, which will include a fasting lipid panel (comprised of total cholesterol, HDL, calculated LDL, and triglycerides) as well as markers of insulin resistance/glycemic dysfunction (C-peptide, insulin, HbA1c) and inflammation (hs-CRP). Moreover, holistic neurodegeneration and neuroaxonal stress will be assessed in all subjects via plasma NfL at baseline and at the post-intervention visit, which will be quantified using electrochemiluminescent immunoassay (ECLIA). In addition, all subjects will be assessed for AD-associated neuropathology at baseline, which will include plasma Aß1–42, Aß1–40, Aß1–42/Aß1–40 ratio, and p-tau217. Aß1–42 and Aß1–40 will be assayed via liquid chromatography-tandem mass spectrometry, the preferred and most accurate approach for amyloid quantification [61]. Furthermore, ECLIA will be utilized to quantify p-tau217. Additional details about how the AD-linked neuropathological biomarkers will be assayed are described elsewhere [62]. All Quest Diagnostics testing locations are appropriately licensed/certified under the Clinical Laboratory Improvement Amendments of 1988 (CLIA 88) and as required by certain State laboratory licensure programs. In addition, Quest Diagnostics’ Regional and Esoteric Reference Laboratories are accredited by the College of American Pathologists.

#### Cognitive assessments

The augmented reality platform delivers a Digital Neuro-Signature (DNS) based on > 700 sensor-derived digital biomarkers captured through brief (10–20 minutes), gamified cognitive tasks that simulate real-world activities [63]. The DNS has demonstrated high sensitivity (≈ 0.91) and specificity (≈ 0.82) in detecting MCI and early AD progression as well as a predictive accuracy of approximately 94% for conversion risk to AD within aging cohorts in prior work [64]. A longitudinal validation study (*N* = 525) showed strong correlations with traditional neuropsychological tests, such as the Mini-Mental State Examination (MMSE) and Montreal Cognitive Assessment (MoCA), and showed that DNS outperformed conventional tools in detecting early cognitive change [64]. The augmented reality-based Cognitive Assessment Platform provides reliable, ecologically valid cognitive phenotyping suitable for remote, high-frequency, digital health studies.

The DNS cognitive assessments, which are suitable for both iPhone iOS and Android devices, are augmented reality technologies that test a participant’s cognitive and functional aptitude via a self-learning (machine learning) algorithm. Cognitive scores will be assessed during baseline and follow-up on site or, for those in the sleep-only intervention group who are physically unable to come on site, at the participants’ homes using an application on a smart device. Participants will be asked to perform tasks using virtual reality representations on each subject’s smartphone’s camera. Using movement and response data plugged into machine learning systems, the service will provide a validated measure of MCI/AD risk, which will be shared with the study team and participants through a computer dashboard and through the app, respectively. The app will test all cognitive domains, but the three following cognitive function outcome variables, which are particularly susceptible to decline in MCI, will be considered the primary cognitive outcomes of interest in this trial: (1) spatial memory, (2) prospective memory, and (3) executive function. All other cognitive functions will be considered secondary outcomes of interest. All data will be automatically transmitted to the study team on an encrypted dashboard. The DNS cognitive assessments have been validated in previous literature [65].

### Statistical analysis plan

#### Power and sample size considerations

This trial is designed to be an exploratory, hypothesis-generating randomized controlled trial. Given the exploratory nature of this three-arm trial, a sample size of 60 participants (20 per group) was selected for this trial to balance feasibility with the ability to detect preliminary signals across intervention arms. Power analyses were conducted via simulation for linear mixed-effects models (LMMs) using estimated parameters relevant to repeated cognitive and biomarker outcomes measured at two time points (baseline (week 0) and 8 weeks). Assuming a moderate effect size (Cohen’s *f*^2^ = 0.25), *α* = 0.05, a compound symmetry covariance structure, and no attrition, this sample size yields approximately 60–65% power to detect a significant group × time interaction. While this power is modest, it is sufficient to estimate effect sizes with reasonable precision and assess trends across intervention arms. Since LMMs maximize available data and accommodate unbalanced designs and missing data, LMMs are preferred over repeated measures ANOVA for small-sample trials with incomplete follow-up. Results from this study will guide power calculations and design refinements for a fully powered randomized controlled trial. As generally recommended for early-phase behavioral intervention studies, this trial’s emphasis will be placed on effect sizes and confidence intervals instead of solely statistical significance.

#### Analysis plan

This study is designed as a three-arm, parallel-group trial comparing the effects of an 8-week exercise intervention (group 1), sleep intervention (group 2), and combined sleep + exercise intervention (group 3) on cognitive performance and biomarkers for brain and cardiometabolic health in older adults with MCI or no cognitive impairment. A total of 60 participants (20 per group) will be enrolled. All analyses will be conducted using IBM SPSS Statistics (Version 31.0.0.0; IBM Corp., Armonk, NY, USA). The primary analytic goal is to determine which intervention arm may demonstrate the greatest improvements in cognitive function and biomarker profiles. Descriptive statistics (means, standard deviations, medians, interquartile ranges, and proportions) will be used to summarize demographic characteristics, intervention adherence, and baseline measures. Primary outcomes will include change**s** in cognitive domain-specific and global cognition scores from the digital cognitive assessments and changes in biomarkers for brain (NfL) and cardiometabolic health (HbA1c, hs-CRP, C-peptide, insulin, total cholesterol, HDL, LDL, triglycerides). Secondary outcomes will include changes in sleep metrics (e.g., sleep efficiency, SWS, REM sleep, and total sleep durations, onset latency, and restlessness, as measured via Elemind) and physical activity/exercise levels (PGE and NPA via Fitbit). Tertiary outcomes will include the role of light exposure (measured via Lys) as a potential modulator of sleep parameters and psychosocial measures (e.g., PROMIS Depression, Social Isolation).

Primary group comparisons will be conducted using LMMs, which offer flexibility for handling repeated measures, unbalanced data, and missingness under the Missing at Random assumption. Each LMM will include fixed effects for group (sleep, exercise, combined sleep + exercise), time (baseline, 8-week follow-up), and the interaction term between group × time. Subject-specific random intercepts will be included to account for intra-individual correlation over time. The main test of intervention efficacy will be the group × time interaction term. To evaluate whether improvements in biomarkers (e.g., HbA1c, hs-CRP) moderate the effects of the interventions on cognitive outcomes, exploratory moderation models will be conducted. These models will include biomarker change scores, group assignment, and group × biomarker interaction terms as predictors of cognitive change. Moderation models will use PROCESS v5.0 Macro for SPSS with bias-corrected bootstrapping (5,000 resamples). Significant interactions will be probed using Johnson-Neyman techniques.

Given the small sample size, power for detecting small effects will be limited. Thus, all models will report estimated effect sizes (Cohen’s *d*, partial *η*^2^), 95% confidence intervals, and significance levels (*α* = 0.05). No corrections for multiple comparisons will be applied to primary outcomes; however, for secondary and exploratory outcomes, a Benjamini–Hochberg false discovery rate (FDR) correction will be used to control for Type I error. Composite indices may be created to reduce variable dimensionality. For cognitive outcomes, domain-specific cognitive function scores will be z-scored and aggregated into a global composite score. For blood-based biomarkers, z-scored values will be averaged to create an overall plasma-based AD risk index as well as a cardiometabolic risk index for all subjects.

Missing data will be handled using maximum likelihood estimation within LMMs. For summary scores or self-report questionnaires with partially missing responses, scale-specific rules will be applied, if available. Participants missing more than 2 weeks of intervention or follow-up cognitive assessments will be excluded from per-protocol models but retained in intention-to-treat analyses. Missingness patterns will be analyzed and classified as Missing Completely at Random (MCAR), Missing at Random (MAR), or Missing Not at Random (MNAR). All assumptions for normality, linearity, and homoscedasticity will be checked using residual plots and Kolmogorov–Smirnov tests. Skewed variables (e.g., hs-CRP) will be square root- or log-transformed prior to modeling. Outliers will be identified using leverage and Cook’s distance, and sensitivity analyses will be performed with and without influential points.

As an alternative analytic strategy, if models fail to yield significant results due to insufficient power or high variability, effect sizes and confidence intervals (CIs) will be emphasized. A simplified analysis approach using ANCOVA (controlling for baseline scores) will be explored for key outcomes. Lastly, pattern mixture modeling may be used to examine intervention effects under different missing data assumptions.

### Ethics and dissemination

All procedures have been approved by the University of Miami Institutional Review Board (IRB) requirements and federal privacy protections and will be conducted in accordance with the Helsinki Declaration. Recruitment and enrollment procedures will comply with all institutional and federal guidelines, including the Health Insurance Portability and Accountability Act (HIPAA), to ensure privacy and confidentiality of all health information obtained. All data will be stored in HIPAA-compliant systems. Fitbit and the Digital Neuro-Signature Platform data will be de-identified and linked using secure participant identification numbers. Results of this randomized controlled trial will be available to scientific researchers and healthcare professionals via publications in peer-reviewed academic journals and presentations at national and international conferences. Data will be made available upon request to the authors upon publication. Furthermore, all scientific as well as the lay public will be able to access study results on the ClinicalTrials.gov website (Identifier: NCT04855630).

### Data and safety monitoring plan

Clinical site monitoring is a vital process that ensures the safety and well-being of human subjects participating in a clinical trial. Clinical site monitoring will be conducted to ensure that the reported trial data is accurate, complete, and verifiable. Site monitoring will also ensure that the trial is in full compliance with the approved protocol/amendment(s), Good Clinical Practices, and applicable regulatory requirements. During the study, a medical doctor will monitor participants who report experiencing medical issues. Should any medical issues arise, participants will be encouraged to seek treatment from the subject’s primary healthcare provider(s) during study enrollment. There are no special considerations for biological data collected by the Quest system, as no biological material will be stored, collected, or analyzed at the University of Miami laboratory. Safety monitoring will include carefully assessing and promptly reporting all adverse events to the appropriate IRBs and Privacy Boards. Medical monitoring will include a regular assessment of the number and type of serious adverse events.

## Discussion

AD remains one of the most pressing public health challenges globally, with MCI as a prodromal stage of the AD continuum that offers a window for preventative intervention. While pharmacological options for slowing AD pathogenesis remain limited, behavioral strategies targeting modifiable lifestyle-based behavioral factors—namely, sleep and exercise—present compelling opportunities for low-risk, scalable, and cost-effective interventions. This study addresses key scientific, clinical, and public health gaps by directly evaluating the independent and combined effects of structured exercise and/or sleep optimization on cognitive, neuropathological, and cardiometabolic outcomes in older adults, including those at elevated risk for neurodegeneration.

From a scientific standpoint, the study seeks to clarify the mechanistic underpinnings by which sleep and/or exercise contribute to cognitive preservation. While existing literature has largely focused on how exercise may improve sleep and subsequently confer neuroprotection, few studies have disentangled the direct versus mediated effects of these behaviors on cognition. Even fewer studies have simultaneously examined how these behaviors interact over time to influence key biological and functional outcomes. By utilizing a three-armed, randomized controlled design, this study offers a unique opportunity to assess whether sleep and exercise operate through overlapping or synergistic pathways in mitigating cognitive decline and altering AD-linked neuropathology, systemic inflammation, and metabolic dysfunction.

Clinically, this study fills a critical gap by targeting a middle-aged and aging population who have greater cognitive vulnerability to AD. The trial moves beyond self-reported behaviors to integrate real-time, ecologically valid assessments of physical activity, sleep, and cognitive function. In addition, the use of the Digital Neuro-Signature Platform, an innovative Food and Drug Administration Breakthrough Device that deploys augmented reality to evaluate cognitive performance in realistic virtual tasks, marks a significant methodological advancement. The augmented reality-based digital cognitive and functional assessment’s ability to simulate real-world scenarios in order to test higher-order cognitive performance, such as memory, navigation, and object placement, offers a nuanced and sensitive measure of cognitive function that surpasses traditional pencil-and-paper tests, especially for detecting early, subtle impairments [64, 66].

Moreover, the study will incorporate light exposure, which will be captured via the Lys device, as a covariate and potential effect modifier. Light is a critical zeitgeber that regulates circadian rhythms, thereby influencing sleep quality and exercise levels [67, 68]. By capturing ambient light exposure, this study will be able to utilize an improved modeling approach to more robustly test how environmental context modulates interventional efficacy and contributes to individual variability in response. This multidimensional measurement strategy will enhance the rigor and ecological validity of the study, essentially intersecting cognitive neuroscience, digital health, and precision prevention.

From a public health perspective, this trial reflects an innovative shift toward digitally enabled, lifestyle-based interventions for cognitive, brain, and cardiometabolic health. Although the exercise intervention utilized in this trial will be conducted in-person to ensure standardization of procedures, the findings may nonetheless inform future hybridized models of care delivery. The inclusion of wearable sensors (Fitbit and Lys) and neuromodulation technology (Elemind) embodies a next-generation toolkit for behavioral clinical trials. These tools will allow for fine-grained, longitudinal monitoring of physiological, behavioral, and cognitive data with minimal participant burden—an essential consideration for older adults, particularly those with cognitive impairment.

Future directions will include the expansion of this trial into a future, fully powered randomized controlled trial to examine long-term cognitive, brain, and cardiometabolic outcomes. Additionally, integrating neuroimaging and more granular sleep staging (e.g., EEG-confirmed SWS) could deepen the mechanistic understanding of how sleep and exercise independently and synergistically influence AD-linked neuropathology and brain function. The study may also open possibilities for tailoring interventions based on individual profiles, such as light exposure patterns, baseline fitness levels, and sleep architecture, to achieve personalized cognitive preservation strategies. In sum, this study represents a novel, multidisciplinary approach that aims to delay or prevent the onset of AD by leveraging the power of wearable technology, augmented reality cognitive testing, and lifestyle interventions. Furthermore, this study responds to an urgent need for scalable, non-pharmacological interventions that can be adapted to diverse populations, laying the foundation for an era of precision behavioral medicine in cognitive aging.

## Supplementary Information

Below is the link to the electronic supplementary material.

ESM1 (DOCX 16.2 KB)

## Data Availability

No data were collected or analyzed for this study. This article describes a study protocol; data will be available upon reasonable request to the corresponding author upon completion of the study.
