# Supplementary material for: Combating Alzheimer’s through sleep and exercise (CASE): A study protocol
Source: GeroScience. 2026 Jun 6;48(5):6353–74. doi: 10.1007/s11357-026-02337-1 (PMC13601473; doi:10.1007/s11357-026-02337-1)
Supplement: Supplementary file 1 [file 11357_2026_2337_MOESM1_ESM.docx]

Dear Roselle Quilala/To Whom it May Concern,

Thank you for your review and prompt feedback on our submission, **JAAA-D-26-00479R1**. However, I wanted to provide some clarification about some of the requests for missing information in case there may have been any confusion in our original submission.

We provided a statement in our original and revised submission already on page **6**. However, for clarification, we have revised the text in the following way in our re-revised submission on page **6**; all changes are highlighted in yellow from our revised submission, but the submission is otherwise verbatim from our first resubmission:

**Consent:** Individuals meeting these pre-screening criteria will then be scheduled for an in-person screening visit to provide written informed consent and further assess eligibility criteria via a seven-day screening period. After signatures are obtained, each prospective subject will be provided with a copy of the signed informed consent document. All procedures were approved by the University of Miami Institutional Review Board (IRB) requirements and federal privacy protections and will be conducted in accordance with the Helsinki Declaration.

(Our inclusion of the Helsinki Declaration was already stated under the "**Ethics and Dissemination**" section on page **19**; however, for more clarification, we have also added this statement immediately after the informed consent statement on page **6**.)

We provided information about funding in our original and first revised submissions under the acknowledgements section after the conclusion section on page **21**. The verbatim funding information is as follows:

**Acknowledgements:** The authors of this work are supported by funding from the National Institutes of Health [R01HL152453], [R01AG075007], [R25HL105444], [R01AG067523], and [R01HL142066]. The funders had no role in the design of the study; in the collection, analyses, or interpretation of data; in the writing of the manuscript; or in the decision to publish the results.

A statement about authorship contributions was already included on the title page of the original and revised submissions on page **1;** the original verbatim text from our initial submissions are as follows:

**Author contributions statement:** All authors contributed to the study conception and design. Specifically, authorship contributions consisted of the following: Conceptualization: Azizi Seixas; Methodology: Azizi Seixas, Debbie Chung, Brittany Larsen, Neva Kirk-Sanchez, Alberto Ramos; Software: Azizi Seixas, Debbie Chung; Resources: Azizi Seixas, Debbie Chung, Girardin Jean-Louis; Writing – Original Draft: Debbie Chung, Brittany Larsen, Azizi Seixas, Laurent G. Garchitorena Gomez, Neva Kirk-Sanchez; Writing – Review & Edit: Debbie Chung, Brittany Larsen, Laurent Garchitorena Gomez, Alberto Ramos, Neva Kirk-Sanchez, Girardin Jean-Louis, Azizi Seixas; Supervision: Brittany Larsen, Neva Kirk-Sanchez; Project administration: Azizi Seixas, Debbie Chung, Brittany Larsen. All authors read and approved the final manuscript.

In response to your request, we have provided a caption for Fig. 1 on page **29** of the main document as follows: Fig. 1. Elemind Neuromodulation Headband.

Please let us know is any further clarification was needed for any of the former three points. Thank you for your time and consideration.
